# What is the empirical evidence that hospitals with higher-risk adjusted mortality rates provide poorer quality care? A systematic review of the literature

**DOI:** 10.1186/1472-6963-7-91

**Published:** 2007-06-20

**Authors:** David W Pitches, Mohammed A Mohammed, Richard J Lilford

**Affiliations:** 1 Department of Public Health and Epidemiology, University of Birmingham, Birmingham, UK

## Abstract

**Background:**

Despite increasing interest and publication of risk-adjusted hospital mortality rates, the relationship with underlying quality of care remains unclear. We undertook a systematic review to ascertain the extent to which variations in risk-adjusted mortality rates were associated with differences in quality of care.

**Methods:**

We identified studies in which risk-adjusted mortality and quality of care had been reported in more than one hospital. We adopted an iterative search strategy using three databases – Medline, HealthSTAR and CINAHL from 1966, 1975 and 1982 respectively. We identified potentially relevant studies on the basis of the title or abstract. We obtained these papers and included those which met our inclusion criteria.

**Results:**

From an initial yield of 6,456 papers, 36 studies met the inclusion criteria. Several of these studies considered more than one process-versus-risk-adjusted mortality relationship. In total we found 51 such relationships in a widen range of clinical conditions using a variety of methods. A positive correlation between better quality of care and risk-adjusted mortality was found in under half the relationships (26/51 51%) but the remainder showed no correlation (16/51 31%) or a paradoxical correlation (9/51 18%).

**Conclusion:**

The general notion that hospitals with higher risk-adjusted mortality have poorer quality of care is neither consistent nor reliable.

## Background

The relationship between quality of care and outcome continues to attract the interests of a wide-spectrum of stakeholders including patients, carers, healthcare providers, researchers, politicians, the media and others [1]. The unit of analysis is often an acute hospital and outcome is frequently defined in terms of a risk-adjusted mortality (inhospital or 30-day). The rationale for using risk-adjusted mortality rates is that they purport to distil the contribution of patient case-mix factors and the play of chance mortality, and thereby expose a residual unexplained variation which may implicate quality of care. This leads naturally to the ranking of hospitals according to risk-adjusted mortality rates with an implied correlation with quality of care [2]. Organisations that produce performance ratings based on mortality rates include Leapfrog [3] and US News "America's Best Hospitals" [4] in the USA, and the Dr. Foster company (the "Good Hospitals" guide [5]), and the Healthcare Commission [6] which uses a "star ratings "system for National Health Service (NHS) hospitals in the UK.

We sought to examine the empirical evidence to clarify the relationship between quality of care and risk-adjusted mortality by undertaking a systematic review which asked the question: " To what extent do hospitals with higher risk-adjusted mortality rates, provide poorer quality of clinical care?"

## Methods

We focused on studies which compared risk-adjusted mortality rates in two or more hospitals and related this to adherence to existing evidence-based standards of clinical care. Evidence of quality of care in our sample of studies was typically obtained from patient case-notes and/or clinical databases ("explicit review ") or expert panels which judged quality of care typically in the form of inspection reports (" implicit review ").

An earlier paper by Iezzoni [7] cited a number of studies that had attempted to answer our research question. Using her paper as a starting point, we identified key words and MEDLINE subject headings (MeSH terms) in these studies. Many contained some of the MeSH terms " process assessment", "outcome assessment", "outcome and process assessment", "quality indicators, health care" and "quality of health care". Most also included "mortality" or "hospital mortality" as a MeSH term, or in the title or abstract.

We applied our search strategy (Additional File 1) to three databases: MEDLINE, CINAHL (Cumulative Index to Nursing and Allied Health Literature) and HealthSTAR (covering health services management literature). We imported references into Reference Manager, version 10 and removed duplicate references.

We included four other papers we were already aware of that met inclusion criteria, but which the database search had not identified. One of these [54] has subsequently been published in a peer-reviewed journal [8]. We scanned the references of all papers and review articles that we obtained, to identify any further studies which might meet the inclusion criteria.

We did not include several types of study : 

(1) Studies that primarily examined the relationship between organisational/structural factors and quality of care (e.g. technical equipment [9], nurse-patient ratio [10], physician staffing [11] or public versus private funding [12,13]) were excluded on the grounds that the underlying evidence-base for such organisational factors is sparse. Moreover, a review of the impact of organisational factors on intensive care outcomes has recently been undertaken [14].

(2) Studies which examined the relationship between volume and outcome, as volume is not an indicator of quality of care, but a structural indicator often associated with quality [15], and the extensive literature on the subject has been repeatedly systematically reviewed [16-18].

(3) Studies where the aim was to discover whether a particular clinical process was effective were excluded, as we were concerned with use of existing knowledge, not the generation of new knowledge [19].

(4) Studies that compared clinical process in one hospital with a clinically equivalent alternative in another [20-22] were also excluded.

(5) Studies that measured quality of care and risk-adjusted mortality but presented insufficient data to enable any conclusions about the nature of the relationship to be drawn [23].

For all studies the present authors independently agreed which papers met the inclusion criteria. Where discrepancies emerged (n = 14 papers) the inclusion/exclusion of these studies was decided by consensus.

For each study we classified the nature of the relationship between quality of care and risk-adjusted mortality as being intuitive (if better care was associated with lower risk-adjusted mortality), no-correlation (if there was no correlation between quality of care and risk-adjusted mortality) and paradoxical (if better care was associated with higher risk-adjusted mortality). It is possible for studies to have more than one relationship, as some studies examined several processes or different clinical conditions.

## Results

Of 6,456 papers located from database searching, initial screening identified 302 papers as meriting further attention, either because titles or abstracts appeared to meet inclusion criteria, or because papers were relevant in another way (e.g. reviews). A further five papers [54-57,59] were located from other sources (e.g. references). On the basis of title or abstract, two of the authors independently selected 91 of these papers to appraise. After applying inclusion and exclusion criteria and agreeing where necessary by consensus, 36 studies remained. One of these was unobtainable [57], but sufficient information was provided in another source [24] for us to be confident that it met inclusion criteria.

Studies were mainly conducted in intensive care units (ICUs) [39,72,73], surgical departments [42-45,57-59,74] or within general medicine [38,41,44,57,58,70]. Conditions most frequently investigated included acute myocardial infarction (AMI) [41,48,50,53-55,57,58,64-69,71], stroke [41,49,51,62], coronary artery bypass graft surgery (CABG) [45,57,58,74] and *Pneumocystis carinii *pneumonia (PCP) [40,60].

There was great diversity in study design, and different studies using the same approach drew conflicting conclusions. Walker [61], using a checklist to review case notes found hospitals with better adherence to processes of care had lower mortality, whereas Dubois [41] did not. Studies of the same condition failed to agree; e.g. for AMI, Keeler [70] found better care in low-mortality hospitals whereas Park [53] found better quality of care in high-mortality hospitals. Results in some studies depended upon the process, so Chen (1) [66] found lower mortality hospitals had higher (better) rates of prescribing of aspirin and β-blockers, but lower (worse) rates of thrombolysis, when compared to high mortality hospitals. Investigating the relationship between mortality and rate of quality-of-care concerns across the USA, Hartz [46] found positive or negative correlation coefficients in different States in the United States of America.

Across the 36 included studies we identified 51 distinct relationships between quality or processes of care and risk-adjusted mortality. Some studies that measured the same process in different settings or subgroups found that the relationship varied according to where it was being measured [e.g. [46]] or how the data was analysed [e.g. [71]] and in such cases we have counted the study more than once in Additional File 4.

Studies which examined the relationship between clinical quality of care variables and risk-adjusted mortality fell into two categories. In most cases (n = 25/36), the authors directly correlated process and risk-adjusted mortality across some or all of the hospitals in the study (Additional File 2) e.g. Dubois [41] undertook case note reviews of patients admitted with stroke, pneumonia and AMI in six high-mortality outlier hospitals and six low-mortality outliers to see whether there was any difference in the quality of care. In eleven studies however, the primary comparison was between hospitals of one sort and hospitals of another (e.g. teaching hospital versus non-teaching hospitals), but both clinical process variables and mortality had been measured (Additional File 3). In these cases the comparison of process and mortality is indirect, for example Gottwik [69] compared clinical processes (aspirin, reperfusion etc) in hospitals with and without cardiology departments, and found that usage was greater (better), and mortality was lower, in hospitals with cardiology departments than those without.

To accommodate the diversity of study design, we analysed the 36 studies in the following ways: (A) direct versus indirect studies; (B) studies grouped by clinical condition; (C) studies grouped by organisations/projects; and (D) studies groups by whether clinical or administrative data was used in risk adjustment (Additional File 4A, 4B and 4C).

### Direct and indirect studies combined

Up to 26 studies provided evidence that better quality of care correlated with lower risk-adjusted mortality rates, which might be considered intuitive. Three of these studies however were only intuitive because of the impact of one outlying hospital, and therefore would have demonstrated no correlation between quality of care and mortality if the outlier hospital was excluded [51,59,62]. Sixteen studies (19 if those studies with only one outlying hospital are included) found no correlation. Nine observed a paradoxical correlation, with better quality in higher risk-adjusted mortality rate hospitals (Additional File 4A).

### Studies grouped by clinical condition(s)

Similarly, to explore whether a relationship between quality of care and risk-adjusted mortality was more commonly observed for specific medical conditions, we analysed studies by condition where applicable, depending upon whether other studies had also analysed these conditions. Additional File 4B shows that approximately half of all studies that were based on a particular type of condition found some degree of positive correlation between better quality of care and lower risk-adjusted mortality; of the others, around two thirds found no correlation and a third found a paradoxical correlation.

### Studies grouped by database or collection of health care units

Some of the above studies could be considered to be non-independent, in that they involve repeated study of the same database or collection of health care units. We identified three such clusters in Additional File 4C: Co-operative Cardiovascular Project studies [64-68,71] (most of which analysed hospitals in different ways using the same clinical dataset); Health Care Financing Administration studies [46,50,53,70] and Veterans Affairs [38,43,44,60] hospitals. The results are not homogeneous in these clusters – we find similar spread between intuitive, paradoxical and null correlation between quality of care and risk-adjusted outcomes.

### Risk-adjustment method

Despite evidence that risk-adjusted mortality is affected by how risk adjustment is undertaken [7,25], only six studies explored the effect of applying different clinical risk adjustment methods. In three cases [50,53,73], effect on mortality was limited; in another, three out of four "high-mortality" hospitals were no longer outliers after accounting for procedure volume [45]. In one study [55,56], hospitals were risk-adjusted for condition of patient on admission and separately for ethnicity, payment method and conditions diagnosed later in admission (that might be caused by poor care). The augmented model reduced the variation in mortality but was compromised by lack of coded information. Only one study, involving five hospitals, found that more extensive risk adjustment further reduced the variation in adjusted mortality rates for stroke patients although one hospital still appeared to have significantly higher risk-adjusted mortality [62].

In our review, some studies used clinical data from hospital records and some used administrative data, collected for example for re-imbursement claims. One may presume that clinical data provides more detailed information for risk adjustment. The proportion of intuitive, null and paradoxical results did not differ according to whether clinical or administrative data were used however [Additional File 4D].

## Discussion

Our systematic review found that the relationship between quality of care and risk-adjusted mortality is inconsistent. Whereas about half the studies reported a positive correlation between quality of care and risk-adjusted mortality, half did not. The notion that mortality can be used to identify poor quality of care stems from a simple function which predicates mortality on three key variables – patient risk-factors (case-mix), play of chance and quality of care. The rationale is that if adequate adjustment for patient case-mix factors (hence risk-adjusted mortality) and the play of chance can be undertaken, then the residual unexplained variance in mortality must be attributable to quality of care. This is a fallacy [26] because it does not acknowledge the role of unmeasured/immeasurable factors in case-mix and how definitions are applied that might affect outcome irrespective of quality. Thus, there are three reasons why outcomes may vary even after case mix adjustment: (i) genuine differences in process measures of quality of care not measured in the study, e.g. vigilance of nursing staff which is harder to measure and therefore rarely captured in the study; (ii) differences in prognosis/risk, not captured in the study; and (iii) differences in definitions or in how definitions were applied in different places.

Furthermore, many studies are prone to Type II error because not every hospital has sufficient patients to ensure that differences in outcomes between units are statistically significant. In reality, even for common operations, only a minority of hospitals actually have sufficient caseloads for even a doubling of the mortality rate to be statistically significant [27].

These factors may explain why in our review we have not found a consistent relationship between quality of care and risk-adjusted mortality.

Nonetheless, even if risk-adjusted mortality rates are affected by quality of care, how well would they perform as a screening tool for poor quality care? A modelling exercise by Hofer [28] in which 10% of hospitals had poor quality care (25% of deaths preventable versus 5% elsewhere) found that sensitivity for detecting poor quality hospitals on the basis of high mortality rates was only 35%, and positive predictive value (PPV) 52%. Mortality for individual medical conditions proved to be an even poorer screening tool (e.g. sensitivity for pneumonia was 10% and PPV 21%, implying that detection via mortality rates would miss 90% of poor-quality hospitals, whilst four out of five hospitals with high risk-adjusted mortality rates had acceptable quality). Similar exercises by Zalkind [29] and Thomas [30] with different input parameters came to the same conclusions.

We found a variety of innovative and complex study designs have been adopted to address the review question and noted no overall consensus over the ideal study design. Further studies should not be undertaken lightly not only because of methodological challenges [26], but also because of the vast quantity of accurate data required. The cost of collecting sufficient data for a risk-adjustment system that would allow fair comparisons of outcomes and quality of care in Californian hospitals was estimated at $61 million in 1990 [31]. An inherent dilemma is that studies which are sufficiently large to detect a significant difference in quality or mortality tend to rely upon administrative databases for clinical data (both for risk-adjusting mortality and measuring quality of care). This is much easier to obtain but may be less reliable than data obtained from manually searching medical records [32,33].

There are several limitations to our review :

We relied upon three medical databases to identify relevant studies and only cited grey literature when either indexed in the databases or referenced in existing studies. The studies that demonstrate a relationship between better quality of care and lower mortality are more likely to be published is essentially un-testable, though it is clear that studies that demonstrate the opposite exist.

Several papers described different aspects of the same study [57,58], or analysed the same data in different ways [66-68] or over different time periods [42,59], meaning that studies were not always independent, although our stratified analyses attempted to control for this.

Unlike reviews of randomised controlled trials, for which comprehensive checklists have been developed to appraise the quality of individual studies [34], no such criteria exists for assessment of quality of care studies. In appraising each study, we had to decide how rigorously it was conducted and how valid its conclusions were. For example, in studies where independent examiners inspected quality of care in high- and low-risk-adjusted mortality hospitals, it was important to find out whether examiners were blind to whether they were visiting a high- or low-risk-adjusted mortality hospital. Where papers discussed previous studies, we noted any comments about their perceived limitations. Where papers initiated correspondence, we looked for letters and authors responses.

Calculated mortality rates vary depending upon the level of detail in the risk adjustment method. Although several studies acknowledged this point, only six studies [45,50,53,55,56,73] recalculated mortality rates applying different risk adjustment techniques. Indeed, one writer sardonically remarked that canny hospitals might even try to calculate their risk-adjusted mortality in different ways and only publish the most favourable result [7]. If identification of a hospital as high-mortality is somewhat arbitrarily dependent upon which method of risk adjustment is used, it is hardly surprising that evidence of a quality-mortality relationship is inconsistent, with some studies "correctly" identifying poor-quality outliers, and others missing poor-quality outliers and identifying false-positives instead. We suggest that future studies comparing risk-adjusted outcomes should include undertake a sensitivity analysis using different risk-adjustment algorithms.

Another important methodological issue is that of hindsight-bias [35]. If peer-review teams visiting hospitals are not blinded, the high mortality rate hospitals may be subject to greater scrutiny because of the case-mix adjustment fallacy [53]. Most studies involving peer reviews stated that reviewers were blind to the mortality status.  This is less problematic when reviewing hospitals but much more challenging when patient case-notes are being reviewed [36].

The definition of mortality was inconsistent. Some studies used inpatient deaths whilst others used death before 30 days or more after admission or after surgery. For example, the identification of outlier hospitals might vary depending on whether all deaths within a defined time period, all hospital deaths attributable to certain conditions or all hospital deaths are counted. Only three studies measured mortality at multiple points. They all found a similar relationship between process and mortality regardless of time [53,64,74].

Studies which attempt to correlate adherence to processes with mortality may be susceptible to ecological fallacy. Some studies used mortality for entire hospitals, but assessed quality of care for specific groups of patients in those hospitals. This may explain why some but not all processes appear to be inversely related to mortality: a hospital could have a low overall mortality yet deliver poor care and have a high mortality rate for patients with AMI; some studies would have considered this a low rather than a high mortality hospital. The degree to which the quality criteria related to measured outcomes was subjective and therefore it was not easy to categorise studies by the degree of fit between process and the outcome that might be affected by that process. In any event, if the quality of care for one type of condition correlates with care in general, and if care in general correlates with outcome, then there should be a correlation between care for one condition and outcomes over many conditions.

Another example of susceptibility to the ecological fallacy is observed in studies where quality of care was not necessarily assessed over the same time period as mortality [46,63]; significant changes in clinical practice could have occurred in the meantime.

We are aware of one other paper that has been published since undertaking our original search [37]. This study found despite significant correlations between risk-adjusted mortality and certain process measures, overall process measures only explained 6% of the variation in hospital mortality rates for patients with AMI.

We suggest that given the consistency of findings across a wide range of different studies that quality of care is only weakly associated with hospital mortality, further research is unlikely to add to the existing body of information. However, there is a need to develop more subtle measures of quality of care, both at the level of patient contact (e.g. vigilance of nursing observations or technical proficiency of surgeons) and at the level of the system (e.g. teamwork and human resources policies).

## Conclusion

Our findings are in agreement with a previous, but not systematic, review [24] of the relationship between quality of care and risk-adjusted mortality. The authors concluded that whilst hospitals that delivered poor-quality care could have higher risk-adjusted mortality rates, hospitals with higher-than-expected risk-adjusted mortality rates did not necessarily provide poor quality care, and different risk-adjusted mortality rates in individual hospitals were not indicative of differences in quality of care.

Despite important methodological concerns, the production of risk-adjustment mortality will almost certainly continue; however logical argument and empirical evidence demonstrates that the link between quality of care and risk-adjusted mortality remains largely unreliable.

## Competing interests

The author(s) declare that they have no competing interests.

## Authors' contributions

RL conceived the idea of the systematic review. DP devised the search strategy and eliminated irrelevant papers. DP and MM independently reviewed the remaining abstracts to decide which to obtain original papers for. Subsequently DP and MM read selected papers and independently decided which met inclusion criteria. In cases where there was disagreement, RL also read the paper and final decision was by consensus. DP wrote the manuscript.

## Pre-publication history

The pre-publication history for this paper can be accessed here:



## Supplementary Material

Additional File 1 Search strategy PDF 11K Click here for file

Additional file 4 Relationship between risk-adjusted mortality and processes of care by several strata – type of correlation, condition and organisation. Bracketed () figures are the most optimistic intuitive count if the three studies from Additional File 2 are included in which the relationship between quality of care and mortality was influenced by one outlier hospital. PDF 17K Click here for file

Additional file 2 Studies that directly correlate processes of care with risk-adjusted mortality PDF 44K Click here for file

Additional file 3 Studies that indirectly correlate process with outcome PDF 29K Click here for file
