# Supplementary material for: What is the empirical evidence that hospitals with higher-risk adjusted mortality rates provide poorer quality care? A systematic review of the literature
Source: BMC Health Serv Res. 2007 Jun 20;7:91. doi: 10.1186/1472-6963-7-91 (PMC1924858; doi:10.1186/1472-6963-7-91)
Supplement: Additional File 1 — Search strategy PDF 11K [file 1472-6963-7-91-S1.pdf]

Additional File 1 Search strategy

Databases
MEDLINE 1966–2005
CINAHL 1982–2005
HealthSTAR 1975–2005

Quality and processes of care search terms
#1. PROCESS ASSESSMENT (HEALTH CARE)/
#2. OUTCOME ASSESSMENT (HEALTH CARE)/
#3. OUTCOME AND PROCESS ASSESSMENT (HEALTH CARE)/
#4. QUALITY INDICATORS, HEALTH CARE/
#5. QUALITY OF HEALTH CARE/

Mortality search terms
#6. mortality.mp.
#7. exp MORTALITY/

Combinations
#8. #1 or #2 or #3 or #4 or #5
#9. #6 or #7
#10. #8 and #9
