# Supplementary material for: What is the empirical evidence that hospitals with higher-risk adjusted mortality rates provide poorer quality care? A systematic review of the literature
Source: BMC Health Serv Res. 2007 Jun 20;7:91. doi: 10.1186/1472-6963-7-91 (PMC1924858; doi:10.1186/1472-6963-7-91)
Supplement: Additional file 4 — Relationship between risk-adjusted mortality and processes of care by several strata – type of correlation, condition and organisation. Bracketed () figures are the most optimistic intuitive count if the three studies from Additional File 2 are included in which the relationship between quality of care and mortality was influenced by one outlier hospital. PDF 17K [file 1472-6963-7-91-S4.pdf]

Additional File 4 Relationship between risk-adjusted mortality and processes of care by several strata – type of correlation, condition and organisation. Bracketed () figures are the most optimistic intuitive count if the three studies from Additional File 2 are included in which the relationship between quality of care and mortality was influenced by one outlier hospital.

**Unit of analysis**	**Type of relationship between risk-adjusted mortality and quality of care**		
	**Intuitive**	**No correlation**	**Paradoxical**
**(A) Type of measurement**			
**Direct**	**15 (18) studies** [39, 41, 43, 44, 45, 46, 47, 50, 53, 54, 55, 57, 58, 61, 63 (51, 59, 62)]	**15 (12) studies** [38, 40, 41, 42, 46, 48, 50, 52, 55, 57, 58, 60, (51, 59, 62)]	**5 studies** [43, 46, 49, 53, 55]
**Indirect**	**8 studies** [64, 65, 66, 68, 69, 70, 71, 73]	**4 studies** [67, 68, 71, 74]	**4 studies** [66, 71, 72, 74]
**Total**	**23 (26) studies**	**19 (16) studies**	**9 studies**
**(B) Clinical Condition**			
**AMI**	**12 studies** [50, 53, 54, 55, 57, 58, 64, 65, 66, 68, 69, 71]	**7 studies** [41, 48, 50, 55, 67, 68, 71]	**4 studies** [53, 55, 66, 71]
**Stroke**	**0 (2) studies** [(51, 62)]	**3 (1) studies** [41, (51, 62)]	**1 study** [49]
**Orthopaedics, trauma, ICU and sepsis**	**2 (3) studies** [39, 73, (59)]	**4 (3) studies** [42, 57, 58, (59)]	**2 studies** [43, 72]
**Dialysis**	**1 study** [47]	**1 study** [52]	**0 studies**
**HIV/PCP**	**0 studies**	**2 studies** [40, 60]	**0 studies**
**(C) Organisation**			
**Co-operative cardiovascular project**	**5 studies** [64, 65, 66, 68, 71]	**3 studies** [67, 68, 71]	**2 studies** [66, 71]
**Health Care Financing Administration**	**4 studies** [46, 53, 70]	**2 studies** [46, 50]	**2 studies** [46, 53]
**Veterans Affairs**	**2 studies** [43, 44]	**2 studies** [38, 60]	**1 study** [43]
**(D) Data source for risk adjustment**			
**Clinical**	**17 (20) studies** [39, 41, 43, 45, 50, 51, 52, 54, 55, 59, 61, 62, 64, 65, 66, 68, 69, 70, 71, 73 (51, 59, 62)]	**12 (15) studies** [40, 41, 42, 48, 50, 53, 55, 60, 67, 68, 71, 74 (51, 59, 62)]	**8 studies** [43, 49, 53, 55, 66, 71, 72, 74]
**Administrative**	**5 studies** [44, 46, 47, 58, 63]	**3 studies** [38, 46, 58]	**1 study** [46]
