# Supplementary material for: What is the empirical evidence that hospitals with higher-risk adjusted mortality rates provide poorer quality care? A systematic review of the literature
Source: BMC Health Serv Res. 2007 Jun 20;7:91. doi: 10.1186/1472-6963-7-91 (PMC1924858; doi:10.1186/1472-6963-7-91)
Supplement: Additional file 2 — Studies that directly correlate processes of care with risk-adjusted mortality PDF 44K [file 1472-6963-7-91-S2.pdf]

Additional File 2 Studies that directly correlate processes of care with risk-adjusted mortality

First author and date of publication	Setting of study	Nature or medical condition(s) of patients	How quality of care was measured	How risk-adjusted mortality was measured	Principal findings	Nature of relationship	Additional comments
Best [38] 1994	Eight Veteran Affairs (VA) hospitals with high mortality and eight with low mortality rates (part of the VA surgical risk study)	Pulmonary disease, gastrointestinal disease, cancer, heart disease, infectious disease	111 deceased patients from high-mortality outliers matched with 111 deceased patients from low-mortality hospitals. Independent reviewers compared quality of care between pairs of patients	30 day mortality; calculated from administrative data, predictive power assessed by c-statistic	No differences between high and low outlier hospitals on preventability of death, and quality of care judged better in the higher-mortality hospitals almost as often as better in the lower mortality hospitals. Results not analysed by disease category.	None	Unable to distinguish between patients with condition at admission and those who developed condition during admission. Investigators blinded to hospital mortality status.
Bulger [39] 2002	34 university hospitals in the US with trauma units	640 adult patients with a head injury and broken arm or leg	Use of prehospital intubation, ICP monitoring, osmotic agents, hyperventilation and CT scan use	In-hospital mortality; calculated from clinical data, predictive power not assessed	Hospitals that undertook more aggressive management had a significantly lower mortality than hospitals that were less aggressive	Intuitive	
Curtis [40] 1997	Two US hospitals	345 HIV-positive patients with PCP	Timeliness of anti-Pneumocystis therapy; use of steroids; diagnosis confirmed by bronchoscopy; use of ICU	In-hospital mortality; calculated from clinical data, predictive power not assessed	Survival not significantly different despite large differences in the rates of diagnostic bronchoscopy and ICU. Differences in use of steroids and antibiotics were not significant.	None	
Dubois [41] 1987	US hospitals: six high-mortality outliers; six low-mortality outliers	378 patients (both alive and who died) admitted with stroke, AMI or pneumonia	(1) Explicit casenote review<br>(2) Implicit casenote review to judge preventability of death	In-hospital mortality; calculated from clinical data, predictive power not assessed	Explicit review: no significant differences between high and low mortality hospitals; implicit review: in high-mortality outliers, significantly more preventable deaths from pneumonia but not stroke or AMI	Explicit: none Implicit: intuitive for pneumonia, none for others	Poor inter-rater reliability;<br>Mortality rates of whole hospitals, not specific conditions;<br>Some physician reviewers were blinded; not clear whether all were
Freeman [42] 2002	Nine hospitals in England in 1997	898 patients aged over 60 years with hip fracture	Timing of surgery and mobilisation, prevention of thromboembolism, geriatrician assessment, use of antibiotics, pressure sore risk assessment	90-day mortality; calculated from clinical data, predictive power not assessed	Significant differences between processes in different hospitals but little variation in outcomes. Comparing 1992 and 1997 [59], some hospitals improved their care whilst others deteriorated. The mortality of the hospital which in 1992 was lower than the others and had better overall care, was no better than the other hospitals by 1997.	None	Follow-up to Todd [59]
Gibbs [43] 2001	Surgical departments in the three highest mortality and five lowest mortality of 44 VA hospitals	739 surgical patients from the VA Surgical Risk Study	Randomly sampled case notes from high and low mortality outlying hospitals; assessing quality of care by (1) structured chart review and (2) in patients who died, review by independent surgeon to estimate preventability of death	30-day mortality; calculated from clinical data, predictive power assessed by c-statistic	Quality of care scores generally slightly lower in high-mortality hospitals, but differences not generally significant. Mean quality of care ratings lower for deceased patients than survivors. Limited evidence that lower mortality associated with better quality of anaesthetic care. Better orthopaedic care associated with higher mortality. Patients at low risk of dying who nevertheless died more likely to have had poor care.	Mixture of intuitive and paradoxical	Moderate inter-rater reliability; not clear whether investigators were blinded to hospital mortality status
Goldman [44] 1994	44 VA hospitals with significantly higher overall or departmental mortality	2,398 deceased acute medical and surgical patients	Peer review of records of dead patients for appropriateness of care and inevitability of death	30-day mortality post-surgery or post-admission; calculated from administrative data, predictive power not assessed	Commonest problem among avoidable deaths was not receiving, or delayed, treatment. Psychiatric units had a significantly higher proportion of avoidable deaths, and proportion with excessive mortality significantly higher than proportion of other hospitals	Intuitive	Began with the 44 highest mortality-rate hospitals rather than comparing high and low-mortality hospitals<br>Poor inter-rater reliability;<br>No mention of blinding to mortality
Hannan [45] 1991	28 New York State hospitals	7,596 patients who underwent open heart surgical procedures	Chart reviews of deceased patients	In-hospital mortality; calculated from clinical data, goodness-of-fit assessed	Rate of quality problems higher in deceased patients from high mortality outliers than low outliers	Intuitive	Only 63 records of deceased patients actually examined; seven records unobtainable. Deaths in one hospital not examined as surgery already suspended. Investigators blinded to mortality
Hartz [46] 1993	4,132 US hospitals (Health Care Financing Administration HCFA study)	2,035,128 Medicare patients in HCFA study	(1) Hospitals ranked in order of peer review organisations' (PROs) problem rates<br>(2) American Hospitals Association survey	30-day mortality; calculated from administrative data, predicted mortality similar to actual mortality	Average within-state Spearman correlation coefficient between problem rate and adjusted mortality rate 0.19 (range -0.32 to 0.29). Similar hospitals had higher correlation coefficients. Significant relationship in 14 states	Predominantly intuitive , though no relationship in some states, paradoxical in a few states	PROs in different states measured quality inconsistently and frequency of quality problems not necessarily correlated with severity of problems. Aggregating at the level of US states may be too course for meaningful results. Paper omitted to present results of hospital survey.
Lowrie [47] 2001	394-498 US haemodialysis units	Renal dialysis patients	Proportion of patients in each unit not conforming to three process indicators (haematocrit, urea reduction ratio, albumin).	Standardised mortality ratios (SMR) from administrative data, evaluated by receiver-operating curves (ROC)	Weak correlation between SMR and variance from guidelines for all three; improvements in indicators unlikely to significantly improve SMR.	Intuitive	Study didn't assess what units were doing to attain process measures;<br>SMR inappropriate for multiple comparisons
Matsui [48] 2001	Four tertiary teaching hospitals in Japan	482 AMI patients	Chart review of a wide range of processes	In-hospital mortality; calculated from clinical data, predictive power not assessed	Similar use of thrombolysis and pacing in all hospitals; wide differences in rates of other procedures and interventions but only one hospital had significantly elevated mortality which was not explained by measured processes.	None	The hospital with the higher mortality may have had sicker patients as not all available data was used to risk-adjust. Chart review preceded establishment of mortality so blinding unnecessary
McNaughton [49] 2003	Three hospitals in New Zealand	181 stroke patients	Adherence to Royal College of Physicians Stroke Audit Package processes of care	12-month mortality; calculated from clinical data	Hospital with poorest process score had lowest mortality; hospital with best process score had highest mortality	Paradoxical	Very long follow-up;<br>23% of stroke patients not included

First author and date of publication	Setting of study	Nature or medical condition(s) of patients	How quality of care was measured	How risk-adjusted mortality was measured	Principal findings	Nature of relationship	Additional comments
Meehan [50] 1995	6 Connecticut hospitals (two from each tertile of mortality)	50 AMI patients in each hospital using HCFA data	Percentage of eligible patients receiving thrombolysis, aspirin and β-blocker	30-day mortality; calculated from clinical data, predictive power assessed by kappa statistic	Percentage thrombolised did not vary between high and low-mortality hospitals; significantly higher percentage receiving aspirin or β-blocker at discharge at low mortality hospitals	Aspirin or β-blocker intuitive; thrombolysis none	
Mohammed [51] 2005	Seven hospitals in the West Midlands, UK	702 stroke patients	Intercollegiate stroke audit package standards	30-day mortality; calculated from clinical data, predictive power not assessed	Widespread differences between hospitals in adherence to processes of care did not, with one exception, correlate with mortality	Intuitive or none (†)	Effects largely the result of poor care in one hospital; missing data limited opportunity to risk adjust
Mozes [52] 1998	7 dialysis units in teaching hospitals in Israel	564 renal dialysis patients	Process variables modifiable by medical staff including intensity of dialysis, laboratory results, albumin, haematocrit, eryothropoetin	1-year mortality; calculated from clinical data, predictive power not assessed	One hospital had elevated mortality, but variations in process indicators could not explain the variation in mortality.	None	
Park [53] 1990	1,121 and 1,137 US hospitals (Health Care Financing Administration study)	2,276 AMI and CCF patients	Explicit review of hospitals with significantly higher or lower than average mortality	In-hospital and 30-day mortality; calculated from clinical data, predictive power not assessed	AMI: quality generally better in higher mortality hospitals. CCF: average quality-of-care score for patients treated in high-mortality hospitals poorer than average score for patients treated in low-mortality hospitals	Paradoxical for AMI, intuitive for CCF	
Peterson [54, 8] 2002	64,775 patients in 350 US hospitals	AMI	% adherence to all relevant American College of Cardiologist/American Heart Association guidelines	In-hospital mortality; calculated from clinical data, predictive power assessed by c-statistic	Comparing quartiles of hospitals ranked by increasing adherence to ACC/AHA process indicators, strong correlation with improving mortality – a 10% improvement in a hospital's composite quality score was associated with a 10% lowering in their patient's odds for mortality	Intuitive	
Romano [55, 56] (California Outcomes Project) 1996	228 Californian hospitals stratified by AMI mortality; ten selected from highest 5%, ten from lowest 5% and ten in middle	AMI patients from each of three strata of hospitals	Use of aspirin, thrombolysis, β blockers, heparin, angiography and revascularisation	In-hospital 30-day mortality; calculated from clinical data	Percentages of patients receiving aspirin did not vary between strata; low-mortality hospitals more likely to prescribe aspirin early. Patients in low-mortality hospitals more likely to have heparin, angiography and revascularisation; patients in high-mortality hospitals more likely to receive β-blockers.	Intuitive for some processes, none or paradoxical for others	Up to 29% of variation in mortality due to coding biases
Thomas [57] 1991	42 US hospitals	Cardiac surgery; AMI; pneumonia; arrhythmia; pelvic and femur fractures	Percentage of cases in each hospital considered by Peer Review Organization review to have quality problems	Condition-specific hospital mortality ratios; study unavailable for further information	Statistically significant correlations for cardiac surgery, AMI and pneumonia, but not arrhythmias and fractures.	Intuitive for some conditions, none for others	Report unavailable (details from review) but frequency of quality problems not necessarily indicative of severity of problems
Thomas [58] 1993	Between 9 and 33 hospitals in Minneapolis (depending on condition being correlated)	Between 1,424 and 8,618 patients with ischaemic heart disease, (including angina, AMI and cardiac surgery), septicaemia	Quality of care judgements by peer-review organisation reviewers	In-hospital mortality; calculated from administrative data, predictive power not assessed	Significant correlation between mortality and quality problem rate for angina, cardiac surgery and AMI; no association for septicaemia	Intuitive for some conditions, none for others	Process measured in 1986-7 and 1987-90, mortality measured 1987-8
Todd [59] 1992	Eight hospitals in England in 1992	580 orthopaedic patients aged over 60 years with hip fracture	Timing of surgery and mobilisation, prevention of thromboembolism, geriatrician assessment, use of antibiotics, pressure sore risk assessment	90-day mortality; calculated from clinical data, predictive power not assessed	Significant differences between processes in different hospitals but little variation in outcomes. One hospital had a significantly lower mortality rate than the others and overall care was better than some of the others though no single process could be identified to account for this.	Intuitive or none (†)	Study repeated by Freeman [42] in 1997 using nine hospitals, seven of which were in the original 1992 study
Uphold [60] 2005	30-34 US hospitals – public, not-for-profit private, for-profit private and VA	1,231 HIV positive patients with PCP, 750 HIV positive patients with community-acquired pneumonia	Timely use of antimicrobial medications and corticosteroids, intubation rates, isolation room, bronchoscopy, chest X-ray utilisation	In-hospital mortality; calculated from clinical data, predictive power not assessed	Rates of bronchoscopy and isolation varied across different categories of hospitals; timeliness of PCP treatments were similar and timelines of community-acquired pneumonia treatment was slower at not-for-profit hospitals than other types of hospitals. Mortality rates were similar across all types of hospitals.	None	
Walker [61] 1988	Five Jamaican hospitals	1,537 dehydrated infants under five	Level of adherence to 26 processes of care	In-hospital, standardised according to dehydration severity from clinical data	Mortality higher in hospitals not conforming to standards of satisfactory care	Intuitive	Each process measure had equal weighting attached
Weir [62] 2001	Five hospitals in Scotland	2,724 stroke patients	Adherence to Royal College of Physicians' Stroke Audit Form processes of care	6-month mortality; calculated from clinical data, predictive power assessed by c-statistic and ROC	One hospital with no specialised stroke service and few patients undergoing CT had much higher mortality than the others. Moderate care differences in other hospitals failed to affect mortality	Intuitive or none (†)	Investigators not blinded<br>Possible systematic difference in how data in non-stroke unit was collected compared to others
Williams [63] 1979	504 hospitals in California	3,441,996 neonates(over 20 weeks gestation up to 28 days post-parturition)	Standards of neonatal intensive care; survey of maternity unit practices	Perinatal; calculated from administrative data, predictive power not assessed	Perinatal mortality lower in hospitals with higher specialist-to-generalist physician ratios and routinely measure Apgar scores	Intuitive	Deaths in 1960 and 1965-1073;<br>Process survey in 1967

(†) In these studies there would have been no correlation between quality of care and mortality had it not been for the existence of one outlier hospital.
