# Supplementary material for: What is the empirical evidence that hospitals with higher-risk adjusted mortality rates provide poorer quality care? A systematic review of the literature
Source: BMC Health Serv Res. 2007 Jun 20;7:91. doi: 10.1186/1472-6963-7-91 (PMC1924858; doi:10.1186/1472-6963-7-91)
Supplement: Additional file 3 — Studies that indirectly correlate process with outcome PDF 29K [file 1472-6963-7-91-S3.pdf]

Additional File 3 Studies that indirectly correlate process with outcome

First author and date of publication	Setting of study	Nature or medical condition(s) of patients	How quality of care was measured	How risk-adjusted mortality was measured	Principal findings	Nature of relationship	Additional comments
Allison [64] 2000	4,361 US hospitals (major and minor teaching, and non-teaching)	114,411 AMI patients over 65 years old in Co-operative Cardiovascular Project	Reperfusion therapy, aspirin, angiotensin-converting enzyme inhibitor (ACEI) at discharge, β-blocker at discharge	30, 60, 90 day and two years after AMI; calculated from clinical data, predictive power assessed by c-statistic	Aggregating hospitals by major, minor and non-teaching status, adherence to processes fell across the three categories and mortality increased	Intuitive .	CCP indicators may have overlooked patients with less strong indications for therapy
Baldwin [65] 2004	4,085 US hospitals (small and large rural, and urban)	135,759 AMI patients over 65 years old in Co-operative Cardiovascular Project	Adherence to all relevant American College of Cardiologist/American Heart Association guidelines	30-day mortality; calculated from clinical data, predictive power not assessed	Adherence to processes increased across different categories of hospitals - remote rural, small rural, large rural and urban, and mortality fell across the four groups. However, despite patients on average being less likely to receive recommended treatments in more rural hospitals, the same proportion of hospitals in each category had almost complete adherence to process guidelines.	Intuitive	
Chen (1) [66] 1999	4,672 US hospitals comparing the 60 highest ranked in "America's Best Hospitals" rating scheme to similarly well-equipped hospitals and others	149,177 AMI patients over 65 years old in Co-operative Cardiovascular Project	Use of aspirin, β-blockers and reperfusion	30-day mortality; calculated from clinical data, predictive power not assessed	Within categories of similar hospitals, mortality varied so widely that many "non-top-ranked" hospitals had lower mortality than the median mortality of the "top-ranked" hospitals'. Significantly more eligible patients were prescribed aspirin or β-blockers in "top-ranked" hospitals, but reperfusion thrombolysis was underutilised. Overall mortality was significantly lower in the 60 "top-ranked" hospitals than similar hospitals not in the "top 60".	Intuitive for aspirin and β-blockers; paradoxical thrombolysis	Mortality measured all cardiovascular deaths, not just AMI . "Best" hospitals were selected not just by mortality but by reputation in the opinion of specialist physicians
Chen (2) [67] 1999	4,672 US hospitals compared those in "100 top hospitals" rating scheme against all others. Same database as Chen (1) above	149,177 AMI patients over 65 years old in Co-operative Cardiovascular Project	Use of aspirin, β-blockers and reperfusion	30-day mortality; calculated from clinical data, predictive power not assessed	Proportion of eligible patients receiving aspirin and β-blockers similar between "top 100 hospitals" and other hospitals. Mortality not significantly different between "top 100" hospitals and others.	None	
Chen (3) [68] 2003	4,221 US hospitals by level of Joint Commission on Accreditation of Healthcare Organisations (JCAHO) accreditation	134,579 AMI patients over 65 years old in Co-operative Cardiovascular Project	Use of aspirin, β-blockers and reperfusion	30-day mortality; calculated from clinical data, predictive power not assessed	Lower proportion of patients admitted to hospitals not surveyed by JCAHO received aspirin, β-blockers or reperfusion and these hospitals had higher than average mortality. Among surveyed hospitals, similar aspirin prescribing regardless of accreditation grade; higher prescribing of β-blockers and reperfusion in better-graded hospitals and lower mortality than overall but differences were modest and within each accreditation level were wide variations in processes and outcomes.	Intuitive between groups, none within groups	JCAHO grading more dependent on managerial factors than clinical<br>Recognised subjectivity in grading a hospital
Gottwik [69] 2001	91 hospitals with cardiology departments and 214 hospitals without cardiology departments in Germany	24,814 AMI patients	Use of aspirin, β-blockers, ACEIs, statins and reperfusion	In-hospital mortality; calculated from clinical data	Reperfusion therapy was used significantly more often in hospitals with cardiology departments, mainly comprising a greater use of primary angioplasty. Hospitals with cardiology departments prescribed aspirin, β-blockers, angiotensin-converting enzyme inhibitors and statins significantly more frequently and had a lower mortality	Intuitive	
Keeler [70] 1993	297 hospitals in five US states (Health Care Financing Administration (HCFA) study)	14,008 patients with CCF, pneumonia, AMI, cerebrovascular disease, hip fracture	(1) Explicit review<br>(2) Implicit review	30-day mortality; calculated from clinical data; predictive power not assessed	Above-average explicit process correlated with above-average implicit process and below-average mortality and vice versa for most hospitals. Overall, teaching hospitals had higher quality scores and lower mortality than rural hospitals. Hospitals with lower process scores had higher mortality, but sample sizes too small to differentiate between individual good and bad hospitals. Results not analysed by disease category.	Intuitive	Investigators aware of which patients died in hospital. Small sample sizes in individual units (5 patients with each condition). Teaching hospitals may have ordered more investigations and recorded more co-morbidities than rural hospitals.
Krumholz [71] 2002	3,363 US hospitals; evaluation of internet rating system which classified hospitals by mortality	141,914 AMI patients in Co-operative Cardiovascular Project	Use of reperfusion, aspirin, β-blockers, ACEIs	30-day mortality; ; calculated from clinical data; predictive power not assessed	Lower mortality associated with hospitals with higher prescribing of aspirin and β-blockers. No trend for ACE-inhibitors. Reperfusion more likely in average or above average mortality hospitals than hospitals with significantly lower than average mortality.	Intuitive for some, none or paradoxical for others in group, none individually	Authors' recalculation of mortality based on partially different timeframe to that of original ratings. Method of risk-adjustment to calculate mortality in star ratings not published by ratings company
Metnitz [72] 2003	31 Austrian ICUs	26,186 intensive care patients aged over 18	Procedures on the Simplified Therapeutic Intervention Scoring System (TISS-28)	In-hospital mortality; calculated from clinical data; predictive power not assessed	Despite greater use of therapeutic interventions than other types of hospitals, teaching hospitals had a significantly higher mortality rate than other types of hospitals	Paradoxical	
Pollack [73] 1991	463 children in 74 hospitals with paediatric intensive care units in Oregon and Washington, USA	Children under 18 years old with head trauma or receiving mechanical ventilation	Processes measured by the Therapeutic Intervention Scoring System (TISS)	Not explicit; calculated from clinical data	Tertiary hospitals had significantly higher TISS scores and significantly lower mortality than other hospitals	Intuitive	Definition of mortality not stated, but presumably is ICU mortality
Ventakappa [74] 2003	13 hospitals in Oklahoma	3,238 CABG patients	Aspirin at discharge; use of internal mammary artery; duration of intubation after CABG	In-hospital and 30-day mortality; calculated from clinical data; predictive power assessed by ROC	Widespread differences between hospitals in adherence to processes of care but only one hospital had significantly higher mortality yet scored significantly better on one of the process indicators. When stratified by volume, hospitals with the lowest mortality had the poorest score on two process indicators.	None or paradoxical	No attempt to correlate process with outcome at the level of individual units, although results of individual units were presented
